# Biological roles of SLC16A1-AS1 lncRNA and its clinical impacts in tumors

**DOI:** 10.1186/s12935-024-03285-6

**Published:** 2024-03-30

**Authors:** Bing Liao, Jialing Wang, Yalin Yuan, Hongliang Luo, Xi Ouyang

**Affiliations:** 1 Department of Otorhinolaryngology, The Second Affiliated Hospital, Jiangxi Medical College, Nanchang University, Nanchang, 330008 Jiangxi China; 2 Department of Gastrointestinal Surgery, The Second Affiliated Hospital, Jiangxi Medical College, Nanchang University, Nanchang, 330008 Jiangxi China; 3 Second School of Clinical Medicine, Jiangxi Medical College, Nanchang University, Nanchang, 330008 Jiangxi China

**Keywords:** SLC16A1-AS1, Human tumor, Tumorigenesis, Cancer biomarker

## Abstract

Recent studies have increasingly highlighted the aberrant expression of SLC16A1-AS1 in a variety of tumor types, where it functions as either an oncogene or a tumor suppressor in the pathogenesis of different cancers. The expression levels of SLC16A1-AS1 have been found to significantly correlate with clinical features and the prognosis of cancer patients. Furthermore, SLC16A1-AS1 modulates a range of cellular functions, including proliferation, migration, and invasion, through its interactions with diverse molecules and signaling pathways. This review examines the latest evidence regarding the role of SLC16A1-AS1 in the progression of various tumors and explores its potential clinical applications as a novel prognostic and diagnostic biomarker. Our comprehensive review aims to deepen the understanding of SLC16A1-AS1’s multifaceted role in oncology, underscoring its potential as a significant biomarker and therapeutic target.

## Introduction

Long non-coding RNAs (lncRNAs) have emerged as pivotal regulators in the complex landscape of cellular biology, gaining significant prominence in cancer research [1–4]. These RNA molecules, exceeding 200 nucleotides and lacking protein-coding capacity [5, 6], were once dismissed as “junk RNAs” [7, 8]. However, the rapid advancement of high-throughput sequencing technologies, such as RNA-Seq and single-cell sequencing techniques, along with progress in bioinformatics in recent years, have led to the identification of an increasing number of lncRNAs [9–14]. Many of these lncRNAs are now known to play vital roles in various physiological processes [15–19]. For example, HOTAIR, one of the earliest reported lncRNAs [20, 21], has been recognized as a significant oncogenic driver [22–25], contributing to tumor cellular signaling transduction [26–28], cancer metabolism reprogramming [29–32], and tumor metastasis [33–36]. Another notable lncRNA, H19, is known for its involvement in embryonic development and imprinting regulation [37–42].

LncRNAs exhibit diverse functions by participating in various cellular processes, such as cell proliferation [43–45], cellular metabolism [46–48], and cellular senescence [49, 50]. And lncRNAs often exhibit dysregulation and are implicated in a range of diseases [51–55], including atherosclerosis [56–58], Alzheimer’s disease [59–61], rheumatoid arthritis [62–64], and particularly in human tumors [65–70]. Notably, lncRNA has emerged as the promising target for the treatment of human diseases [71–73]. Especially with the development of CRISPR/Cas9 technology, lncRNA genes can be precisely manipulated to study their role in disease [74–77]. This helps identify new lncRNAs that are expected to become new targets and biomarkers for cancer treatment. Furthermore, the use of CRISPR/Cas9-based screens to discover lncRNAs involved in drug resistance also opens avenues for the development of more effective therapeutic strategies [78]. These advances underscore the importance of lncRNA in precision medicine and cancer treatment and mark an important step forward for customized medical solutions.

LncRNAs are broadly categorized into several types based on their genomic locations, encompassing intronic, intergenic, and antisense lncRNAs [79–82]. LncRNAs play crucial roles in regulating gene expression at multiple levels, including chromatin modification, transcription, and post-transcriptional processing [83–88]. Among these, antisense lncRNAs have recently garnered attention since their critical role in the tumor development [89, 90]. Antisense lncRNAs are types of long noncoding RNA molecules that are transcribed from the DNA strand opposite to the sense strand [91]. These RNAs can regulate gene expression through various mechanisms [89, 90, 92], including base pairing with sense RNAs, affecting their stability, translation, and splicing, or by recruiting chromatin-modifying enzymes to specific genomic regions. Their actions contribute to complex regulatory networks within cells, influencing numerous biological processes and disease states.

SLC16A1 antisense RNA 1 (SLC16A1-AS1) is a novel antisense lncRNA which has become a rising star in oncological research. SLC16A1-AS1 exhibits aberrant expression in a variety of cancers, including glioblastoma (GBM) [93, 94], oral squamous cell carcinoma (OSCC) [95, 96], hepatocellular carcinoma (HCC) [97–100], renal cell carcinoma (RCC) [101], bladder cancer [102], cervical squamous cell carcinoma(CSCC) [103], breast cancer [104–106], osteosarcoma [107], and non-small cell lung cancer (NSCLC) [108]. Furthermore, SLC16A1-AS1 exhibits multiple biological roles in these primary malignancies, highlighting its complexity in tumorigenesis and its potential as a tumor biomarker [93–108]. SLC16A1-AS1 is intricately linked to the proliferation, migration and invasion of tumor cells, and its abnormal expression is also related to the clinical characteristics and prognosis of cancer patients [93–108].

This article comprehensively reviews the latest findings on the role of SLC16A1-AS1 in a spectrum of human cancers, delving into its expression patterns and molecular mechanisms across different cancer types, and evaluating its viability as a prognostic and diagnostic marker. This review aims to enrich our understanding of the multifaceted role of SLC16A1-AS1 in oncology, highlighting its promise as a key biomarker and therapeutic target.

### Functional roles of SLC16A1-AS1 in different tumors

SLC16A1-AS1, a tumor-associated lncRNA recently uncovered, has exhibited dysregulated expression patterns across a range of cancer types, garnering considerable interest for its potential involvement in tumor development. In vitro and in vivo investigations have illuminated the multifaceted roles of SLC16A1-AS1 in oncogenesis. These studies, utilizing an array of cell-based assays, have probed its impact on crucial cellular activities, including proliferation, apoptosis, cell cycle regulation, migration, and invasion. Table 1 summarizes the expression patterns of SLC16A1-AS1 in various tumors, highlighting their relevant functional effects and roles in cancer progression. The subsequent sections explore in detail the specific functions and regulatory mechanisms of SLC16A1-AS1 in different tumors.

**Table 1:** Experimental role of SLC16A1-AS1 in various cancer types

Tumor type	Experiment	Regulatory mechanism	Biological function	Role	Ref.
Glioblastoma	In vitro	SLC16A1-AS1/miR-1269 (mature and premature)	Proliferation, apoptosis, migration, and invasion	Oncogene	[93]
	In vitro	SLC16A1-AS1/miR-149 methylation	Proliferation	Oncogene	[94]
Oral squamous cell carcinoma	In vitro	SLC16A1-AS1/miR-5088-5p (mature and premature)	Proliferation	Tumor suppressor	[95]
	In vitro	/	Proliferation, cell cycle arrest	Oncogene	[96]
Hepatocellular carcinoma	In vitro	SLC16A1-AS1/miR-141 methylation	Proliferation	Oncogene	[97]
	In vitro	SLC16A1-AS1/miR-301b-3p/CHD5	Cell viability, proliferation, apoptosis, invasion, radiosensitivity, and EMT	Tumor suppressor	[98]
	In vitro	SLC16A1-AS1/miR-­411/MITD1	Cell viability, proliferation, cell migration and invasion	Oncogene	[99]
Renal cell carcinoma	In vitro	SLC16A1-AS1/miR-143-3p/SLC7A11	Cell viability, proliferation, migration and ferroptosis	Oncogene	[101]
Bladder cancer	In vitro	E2F1/SLC16A1-AS1/MCT1	Metabolic reprogramming, invasion, and proliferation	Oncogene	[102]
Cervical squamous cell carcinoma	In vitro	SLC16A1-AS1/miR-194/SOCS2	Proliferation	Tumor suppressor	[103]
Breast cancer	In vitro	MiR526b/SLC16A1-AS1	Proliferation, apoptosis and invasion	Oncogene	[104]
	In vitro and in vivo	SLC16A1-AS1/miR-552-5p/WIF1	Cell viability, proliferation, migration, invasion, and tumor growth	Tumor suppressor	[105]
	In vitro	SLC16A1-AS1/miR-182/PDCD4	Proliferation, and cell cycle arrest	Tumor suppressor	[106]
Non-small cell lung cancer	In vitro	RAS/RAF/MEK pathway	Cell viability, proliferation, apoptosis, and cell cycle arrest	Tumor suppressor	[108]

### Glioblastoma

In glioblastoma, a notably aggressive form of brain tumor [109–111], SLC16A1-AS1 has been identified as playing a pivotal oncogenic role, as elucidated by in vitro studies. Research by Jin et al. [93] revealed that in glioblastoma cells, the lncRNA SLC16A1-AS1 regulates both mature and premature forms of miR-1269. This regulation is linked to significant changes in cancer cell behaviors, including proliferation, apoptosis, migration, and invasion, indicating SLC16A1-AS1’s role as an oncogene. Furthermore, Long et al. [94] complemented these findings by demonstrating that SLC16A1-AS1 is upregulated in glioblastoma and influences cancer cell proliferation through the epigenetic modification, specifically the methylation of miR-149. This interaction between SLC16A1-AS1 and miR-149 methylation further cements the role of SLC16A1-AS1 as an oncogene in glioblastoma. The distinct expression patterns and functional impacts of SLC16A1-AS1, involving crucial processes like microRNA regulation and methylation, not only advance our understanding of glioblastoma’s molecular mechanisms but also highlight the potential of SLC16A1-AS1 as a biomarker for glioblastoma, providing promising avenues for targeted cancer therapies and diagnostic strategies.

### Oral squamous cell carcinoma

SLC16A1-AS1 plays a nuanced role in the pathogenesis of OSCC. Li et al. [95] delves into the interaction between SLC16A1-AS1 and miR-5088-5p (both mature and premature forms) in OSCC. This study indicates that SLC16A1-AS1 acts as a tumor suppressor by modulating the behavior of miR-5088-5p, which in turn affects cancer cell proliferation [95]. The specific mechanism through which SLC16A1-AS1 exerts this suppressive effect, particularly in relation to miR-5088-5p, highlights a complex interplay that could significantly influence OSCC progression.

In contrast, the findings of Feng et al. [96] portray SLC16A1-AS1 in a different light. This study does not specify an interaction with a particular miRNA but focuses on the broader tumorigenesis features of SLC16A1-AS1 in OSCC. Here, SLC16A1-AS1 is seen to contribute to cancer cell proliferation and cell cycle arrest, suggesting its role as an oncogene [96]. This oncogenic aspect of SLC16A1-AS1 in OSCC points to its potential involvement in accelerating cancer progression by disrupting normal cell cycle regulation.

These contrasting studies collectively indicate that SLC16A1-AS1 has a complex and dualistic role in OSCC. On one hand, it interacts with specific miRNAs like miR-5088-5p, potentially acting as a tumor suppressor. On the other hand, it exhibits characteristics of an oncogene, influencing cell proliferation and cell cycle processes. This duality underscores the intricate nature of SLC16A1-AS1’s involvement in OSCC and suggests that its role may vary depending on the molecular context and cellular environment. Understanding these dynamics is crucial for developing targeted therapeutic strategies for OSCC.

### Hepatocellular carcinoma

SLC16A1-AS1 exhibits a complex and significant role in HCC [97–99]. Tian et al. [97] reported that SLC16A1-AS1 is upregulated in HCC and is associated with poor patient survival, positioning it as a potential prognostic biomarker. Furthermore, SLC16A1-AS1’s interaction with miR-141 through methylation has been shown to promote cell proliferation, characterizing it as an oncogene. This oncogenic nature is further supported by the study of Duan et al. [99], which demonstrates that SLC16A1-AS1 modulates the miR-411/MITD1 axis, affecting HCC cell viability, proliferation, migration, and invasion.

Conversely, Pei et al. [98] presented a contrasting role of SLC16A1-AS1 in HCC. Their findings suggest that SLC16A1-AS1 acts as a tumor suppressor by regulating the miR-301b-3p/CHD5 axis, impacting cell viability, proliferation, apoptosis, invasion, radiosensitivity, and the epithelial-mesenchymal transition (EMT) process. This suppression indicates a therapeutic potential for SLC16A1-AS1 in enhancing the efficacy of radiotherapy for HCC and controlling cancer progression.

These diverse findings collectively underscore the multifaceted nature of SLC16A1-AS1 in HCC. Its ability to act both as an oncogene and a tumor suppressor, depending on its interactions with specific microRNAs and the resulting cellular effects, highlights its crucial involvement in the molecular mechanisms of HCC. This dualistic nature of SLC16A1-AS1 offers insightful perspectives into potential therapeutic interventions, targeting different aspects of its function to improve treatment outcomes and patient survival in HCC.

### Renal cell carcinoma

In renal cell carcinoma, SLC16A1-AS1 has been identified as playing a critical oncogenic role, as demonstrated in an in vitro study by Li et al. [101]. This study demonstrates that SLC16A1-AS1 interacts with the miR-143-3p/SLC7A11 signaling pathway, impacting crucial aspects of cancer cell behavior. These aspects include cell viability, proliferation, migration, and ferroptosis, an iron-dependent form of cell death [112, 113]. Specifically, the silencing of SLC16A1-AS1 induces ferroptosis in renal cell carcinoma cells [101]. This is mediated through an increase in miR-143-3p levels, leading to the subsequent downregulation of SLC7A11, a critical regulator of ferroptosis [114–116]. The normal function of SLC16A1-AS1 in renal cell carcinoma appears to be the suppression of ferroptosis, thereby promoting cell survival and proliferation. This oncogenic action of SLC16A1-AS1, through its interaction with miR-143-3p and SLC7A11, highlights its significant role in the progression and survival of renal cell carcinoma. The study underscores the potential of targeting SLC16A1-AS1 in therapeutic strategies, where manipulating its expression could promote ferroptosis in cancer cells, offering a novel approach to treat renal cell carcinoma.

### Bladder cancer

In bladder cancer [102], SLC16A1-AS1 plays a significant role in cancer metabolism, closely involved in tumor progression. SLC16A1-AS1 functions both as a target and a co-activator of the transcription factor E2F1, which is known to be involved in various cellular processes [117–121], including cell cycle regulation and apoptosis. The interaction between SLC16A1-AS1 and E2F1 in bladder cancer leads to changes in the metabolic pathways of the cancer cells, a process termed metabolic reprogramming [122–124]. This reprogramming is essential for cancer cells to meet the increased energy and biosynthesis demands for rapid growth and proliferation [125–127]. The involvement of SLC16A1-AS1 in this process underscores its role in supporting the metabolic needs of rapidly dividing cancer cells. Furthermore, the study suggests that SLC16A1-AS1, through its interaction with E2F1, contributes to the progression of bladder cancer [102]. It may influence the expression of genes involved in metabolism, thereby facilitating the altered metabolic state that is characteristic of cancer cells. In a word, SLC16A1-AS1 in bladder cancer is implicated in several key aspects of cancer progression, particularly through its role in metabolic reprogramming, invasion, and proliferation, mediated by its interaction with E2F1 and MCT1. Understanding this intricate molecular interplay offers valuable insights into potential therapeutic targets, focusing on disrupting these crucial metabolic and proliferative pathways in bladder cancer.

### Cervical squamous cell carcinoma

In CSCC, SLC16A1-AS1 functions as a key regulator in cancer cell biology, particularly influencing cell proliferation [103]. The study revealed that SLC16A1-AS1 inhibits cell proliferation in CSCC [103]. This suppression is mediated through the miR-194/SOCS2 axis, indicating a complex interaction between the lncRNA, microRNA, and downstream signaling molecules. miR-194 is known to play a role in various cellular processes and is involved in cancer progression [128–130]. In CSCC, SLC16A1-AS1 appears to regulate the expression or activity of miR-194, which in turn impacts the expression of SOCS2. And SOCS2 has been reported to participate in multiple cellular pathways and regulate tumor proliferation [131–134]. The downregulation of miR-194 by SLC16A1-AS1 leads to increased expression of SOCS2, which contributes to the suppression of cell proliferation.

This study sheds light on the tumor-suppressive role of SLC16A1-AS1 in CSCC, contrasting with its role in other cancer types where it may function as an oncogene. The SLC16A1-AS1/miR-194/SOCS2 axis provides a novel insight into the molecular mechanisms underlying CSCC, suggesting that modulation of this axis could be a potential therapeutic strategy in treating this type of cancer. By targeting this pathway, it may be possible to control or reduce the proliferation of CSCC cells, offering a promising avenue for future cancer therapies.

### Breast cancer

In breast cancer (BC), and particularly in the context of triple-negative breast cancer (TNBC), SLC16A1-AS1 emerges as a key player, exhibiting diverse roles [104–106]. The research conducted by Zhao et al. [104] reported an interaction between miR-526b and SLC16A1-AS1, which significantly affects breast cancer cell proliferation, apoptosis, and invasion. This interaction suggests that miR-526b acts as an oncogene by targeting SLC16A1-AS1, highlighting a complex regulatory mechanism in TNBC. However, the study by Jiang et al. [105] demonstrates both in vitro and in vivo that the overexpression of SLC16A1-AS1 suppresses cell viability, proliferation, migration, invasion, and tumor growth in breast cancer through the miR-552-5p/WIF1 signaling pathway. This finding underscores the tumor-suppressive role of SLC16A1-AS1, which contrasts with its interaction with miR-526b and suggests a multifaceted function in different contexts of breast cancer. Additionally, Jiang et al. [106] report that SLC16A1-AS1 regulates the miR-182/PDCD4 axis, affecting cell proliferation and inducing cell cycle arrest in TNBC. This regulatory action further confirms the tumor-suppressive role of SLC16A1-AS1 in breast cancer, particularly in controlling the cell cycle and proliferation of TNBC cells. These studies collectively highlight the complex and dualistic role of SLC16A1-AS1 in breast cancer. Its interaction with different miRNAs can lead to either oncogenic or tumor-suppressive outcomes, affecting key cancer cell behaviors like proliferation, apoptosis, invasion, and tumor growth. The diverse impacts of SLC16A1-AS1 in breast cancer, particularly in TNBC, point to its potential as a multifaceted target for therapeutic interventions, with the possibility of manipulating its expression or function to impede cancer progression.

### Non-small cell lung cancer

In NSCLC, in vitro studies conducted by Liu et al. [108] have identified SLC16A1-AS1 as a significant contributor to NSCLC progression. SLC16A1-AS1 plays a role in modulating the RAS/RAF/MEK signaling pathway, which is pivotal in cell growth and survival [135–137]. This modulation characterizes SLC16A1-AS1 as a tumor suppressor, impacting crucial cellular processes such as cell viability, proliferation, apoptosis, and cell cycle arrest in NSCLC [108]. The interaction of SLC16A1-AS1 with this signaling pathway highlights its involvement in the cellular mechanisms governing cancer progression, particularly in regulating cell growth and apoptosis. The functional significance of SLC16A1-AS1 in NSCLC underscores its importance not only in elucidating the molecular intricacies of the disease but also in its potential for targeted therapeutic strategies. Investigating the multifaceted roles of SLC16A1-AS1 could pave the way for innovative treatments and management strategies in NSCLC, potentially enhancing patient outcomes.

### Prognostic and diagnostic values of SLC16A1-AS1 in different tumors

SLC16A1-AS1 has emerged as a significant biomarker with diverse clinical implications and prognostic and diagnostic values across different types of cancers (Table 2). In glioblastoma [93, 94], SLC16A1-AS1 consistently exhibits upregulation and is closely associated with key clinical features such as tumor size and stage, ultimately impacting overall survival. This upregulation pattern is consistently observed in 3 out of 4 studies on HCC [97–100]. Elevated expression of SLC16A1-AS1 in HCC samples is strongly linked to poor prognosis, including adverse outcomes in overall survival, progression-free survival, and distant metastasis-free survival [100]. Notably, Song et al. [100] demonstrated that SLC16A1-AS1 shows high sensitivity and specificity in predicting both survival and the likelihood of distant metastasis in HCC patients. Conversely, in OSCC, SLC16A1-AS1 exhibits variable expression, being both upregulated and downregulated in different studies [95, 96], with implications on histologic grades and overall survival. This variability underscores the intricate nature of SLC16A1-AS1’s role in cancer biology.

Expanding its clinical relevance, SLC16A1-AS1 displays upregulation in the stromal tissue of PDAC compared to the tumor epithelium [138]. This suggests a potential role for SLC16A1-AS1 in the remodeling of the extracellular matrix within the tumor environment. In colorectal cancer [139, 140], the upregulation of SLC16A1-AS1 in cancer tissues is significantly associated with BRAF mutation and overall survival. Additionally, it exhibits diagnostic potential in distinguishing tumor tissue from normal tissue.

In renal cell carcinoma and bladder cancer, SLC16A1-AS1 has shown diagnostic potential, particularly in bladder cancer [102], where a high AUC value suggests its effectiveness in bladder cancer diagnosis. Moreover, its upregulation is linked to poorer overall survival in renal cell carcinoma patients [101, 141].

Breast cancer presents a unique scenario where SLC16A1-AS1 exhibits a complex expression pattern, including both upregulation and downregulation in cancer tissues [104–106, 142]. When downregulated, it is associated with advanced clinicopathologic characteristics such as larger tumor size, higher TNM stage, and the presence of lymph node metastasis [105]. Furthermore, this downregulation correlates with unfavorable prognosis, including reduced overall survival and disease-free survival [105]. Additionally, the downregulation of SLC16A1-AS1 in the plasma of breast cancer patients suggests its potential as a circulating biomarker for diagnostic or prognostic purposes [142].

In endometrial and cervical squamous cell carcinoma, SLC16A1-AS1’s upregulation and downregulation respectively are associated with overall survival [103, 143], suggesting its role in prognostication in these cancers. Furthermore, in NSCLC [108], downregulation of SLC16A1-AS1 is linked to patient demographics, disease progression, and survival outcomes, highlighting its prognostic significance.

**Fig. 1:**
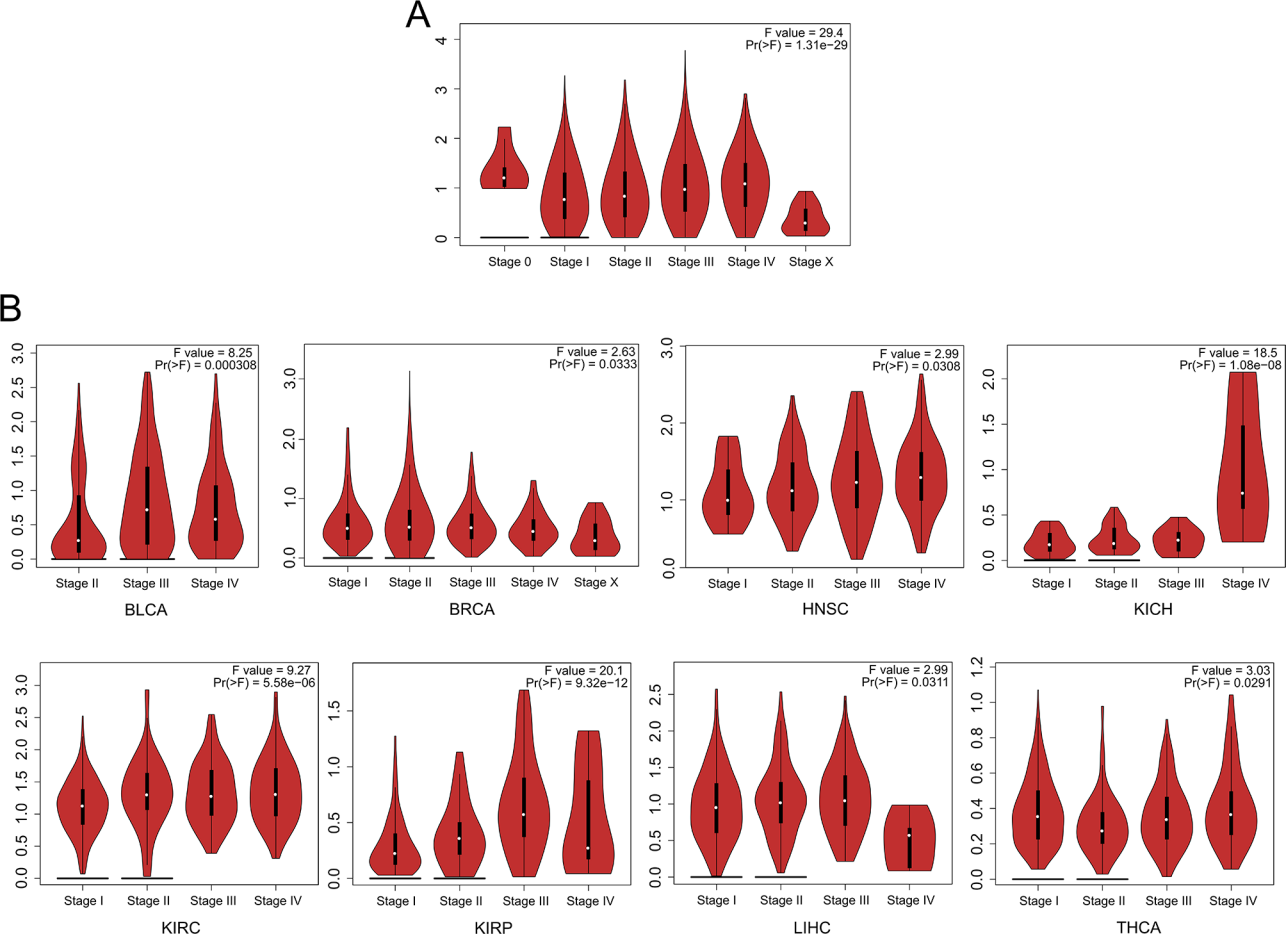
Association Between SLC16A1-AS1 Expression Levels and Pathological Stages of Human Tumors. (**A**) Meta-analysis of pathological staging data across 26 different types of tumors, providing insights into the correlation between SLC16A1-AS1 expression levels and cancer progression. (**B**) Significant associations in eight tumor types: Bladder Urothelial Carcinoma (BLCA), Breast Invasive Carcinoma (BRCA), Head and Neck Squamous Cell Carcinoma (HNSC), Kidney Chromophobe (KICH), Kidney Renal Clear Cell Carcinoma (KIRC), Kidney Renal Papillary Cell Carcinoma (KIRP), Liver Hepatocellular Carcinoma (LIHC), and Thyroid Carcinoma (THCA). This panel highlights specific tumor types where a significant association exists between SLC16A1-AS1 expression levels and pathological staging, offering valuable insights into the diverse roles of SLC16A1-AS1 across various cancer contexts

Recognizing the pivotal role of pathological staging in prognosis assessment, we conducted a comprehensive analysis using GEPIA2 (http://gepia2.cancer-pku.cn/#index) [144], a prominent online tool for gene expression analysis in cancer, to investigate the correlation between SLC16A1-AS1 expression levels and pathological staging across various tumors. Our initial meta-analysis of pathological staging data revealed a significant relationship between SLC16A1-AS1 expression levels and clinical stage in human tumors (Fig. 1A). Further analysis identified a significant association between SLC16A1-AS1 expression levels and pathological staging in eight specific cancer types: Bladder Urothelial Carcinoma (BLCA), Breast Invasive Carcinoma (BRCA), Head and Neck Squamous Cell Carcinoma (HNSC), Kidney Chromophobe (KICH), Kidney Renal Clear Cell Carcinoma (KIRC), Kidney Renal Papillary Cell Carcinoma (KIRP), Liver Hepatocellular Carcinoma (LIHC), and Thyroid Carcinoma (THCA) (Fig. 1B).

Overall, SLC16A1-AS1 serves as a multifaceted biomarker across various cancer types, playing a significant role in prognosis, with potential applications in diagnosis and patient stratification. The diverse expression patterns of SLC16A1-AS1 across different cancers underscore the complexity of its function in oncology and the importance of context-specific evaluations for its application in clinical practice.

**Table 2:** Abnormal expression of SLC16A1-AS1 and its clinical significance in different cancers

Tumor type	Expression	Clinical features	Prognosis	Diagnosis	Ref.
Glioblastoma	Upregulated in cancer tissues	Tumor size	/	/	[93]
	Upregulated in cancer tissues	Clinical stage, KPS score	OS	/	[94]
Oral squamous cell carcinoma	Downregulated in cancer tissues	Tumor size	/	/	[95]
	Upregulated in cancer tissues	Histologic grade	OS	/	[96]
Hepatocellular carcinoma	Upregulated in cancer tissues	/	OS	/	[97]
	Downregulated in cancer tissues	/	OS	/	[98]
	Upregulated in cancer tissues	/	OS, PFS, and DmFS	AUC (OS):0.785, AUC(PFS): 0.808, AUC(DmFS): 0.764	[100]
	Upregulated in cancer tissues	/	OS	/	[99]
Pancreatic ductal adenocarcinoma	Upregulated in PDAC stroma than in the epithelium	/	/	/	[138]
Colorectal cancer	Upregulated in cancer tissues	BRAF mutation	/	AUC: 0.668	[139]
	Upregulated in cancer tissues	/	OS	**AUC: 0.88**	[140]
Renal cell carcinoma	Upregulated in cancer tissues	/	/	/	[145]
	Upregulated in cancer tissues	/	OS	/	[101]
	Upregulated in cancer tissues	/	/	AUC: 0.6022	[146]
	Upregulated in cancer tissues	/	OS	/	[141]
Bladder cancer	Upregulated in high grade tumor tissues	/	/	AUC: 0.923	[102]
	Upregulated in cancer tissues	/	/	/	[147]
Endometrial carcinoma	Upregulated in cancer tissues	Clinical stage, histological grade	OS	/	[143]
Cervical squamous cell carcinoma	Downregulated in cancer tissues	/	OS	/	[103]
Breast cancer	Upregulated in cancer tissues	/	/	/	[104]
	Downregulated in cancer tissues	Tumor size, TNM, LNM	OS, DFS	/	[105]
	Downregulated in cancer tissues	/	/	/	[106]
	Downregulated in plasma of breast cancer	/	/	/	[142]
Osteosarcoma	Upregulated in cancer tissues	/	/	/	[107]
Non-small cell lung cancer	Downregulated in cancer tissues	Sex, smoking, vital status	OS, PFS	/	[108]

OS: Overall survival; PFS: Progression-free survival; DmFS: Distant metastasis-free survival; DFS: Disease-free survival; TNM: Tumor, Node, Metastasis; LNM: lymph nodes metastasis

### Future perspectives

**Fig. 2:**
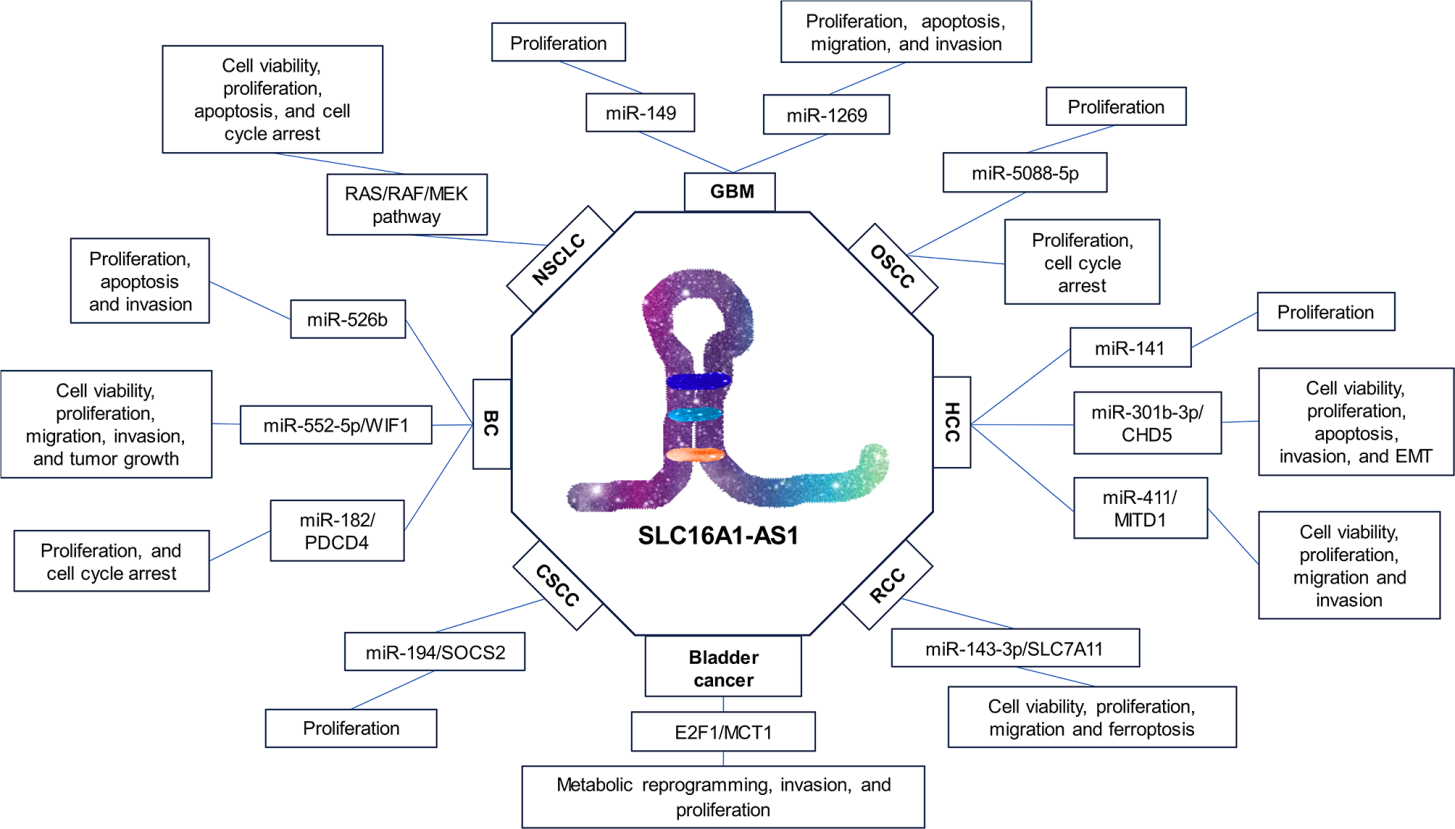
Regulatory Mechanisms Associated with SLC16A1-AS1 and Its Role in Different Cancers. This diagram features SLC16A1-AS1 at the center, prominently highlighted within a hexagonal frame to symbolize its central role in various cancers. Surrounding this central hexagon, rectangles represent the potential regulatory and functional interactions of SLC16A1-AS1 with various cellular processes and pathways in cancer. Each rectangle is designed to contain specific details of mechanisms of action, signaling molecules, microRNAs, or proteins that are influenced by or interact with SLC16A1-AS1. This layout underscores the complexity and multifaceted nature of SLC16A1-AS1’s impact on cellular behavior and cancer pathogenesis

SLC16A1-AS1, a complex and multifunctional lncRNA, has become a focal point in cancer research, as depicted in Fig. 2. This underscores the necessity for comprehensive research into its molecular mechanisms across a range of cancer types. SLC16A1-AS1 exerts a substantial influence on crucial cell behaviors, including cell viability, proliferation, apoptosis, cell cycle regulation, migration, invasion, tumor growth, ferroptosis, and metabolic reprogramming. Its dual functionality as either an oncogene or a tumor suppressor, depending on the cancer type and stage, highlights the importance of fully understanding its varied roles and regulatory pathways within the complex framework of cancer biology.

However, the precise regulatory mechanisms of SLC16A1-AS1 in cancer remain elusive. Investigating its interactions with additional signaling pathways, microRNAs, and proteins is crucial for gaining a comprehensive understanding. In-depth mechanistic studies, particularly those involving in vivo research, are essential to deepen our understanding of SLC16A1-AS1’s role in tumor initiation and progression. It is also necessary to assess SLC16A1-AS1’s distinct functions and regulatory mechanisms at both the early and late stages of cancer progression. Importantly, the aberrant expression of SLC16A1-AS1, which influences malignant behaviors in tumors, suggests that targeting its expression by small molecule inhibitors, antisense oligonucleotides, or CRISPR/Cas9-based approaches could unveil new therapeutic opportunities. Moreover, SLC16A1-AS1 is implicated in the regulation of ferroptosis and metabolic reprogramming, both of which are associated with tumor drug resistance [32, 148–150]. Therefore, investigating the role of SLC16A1-AS1 in therapeutic resistance presents a promising avenue for future research.

**Fig. 3:**
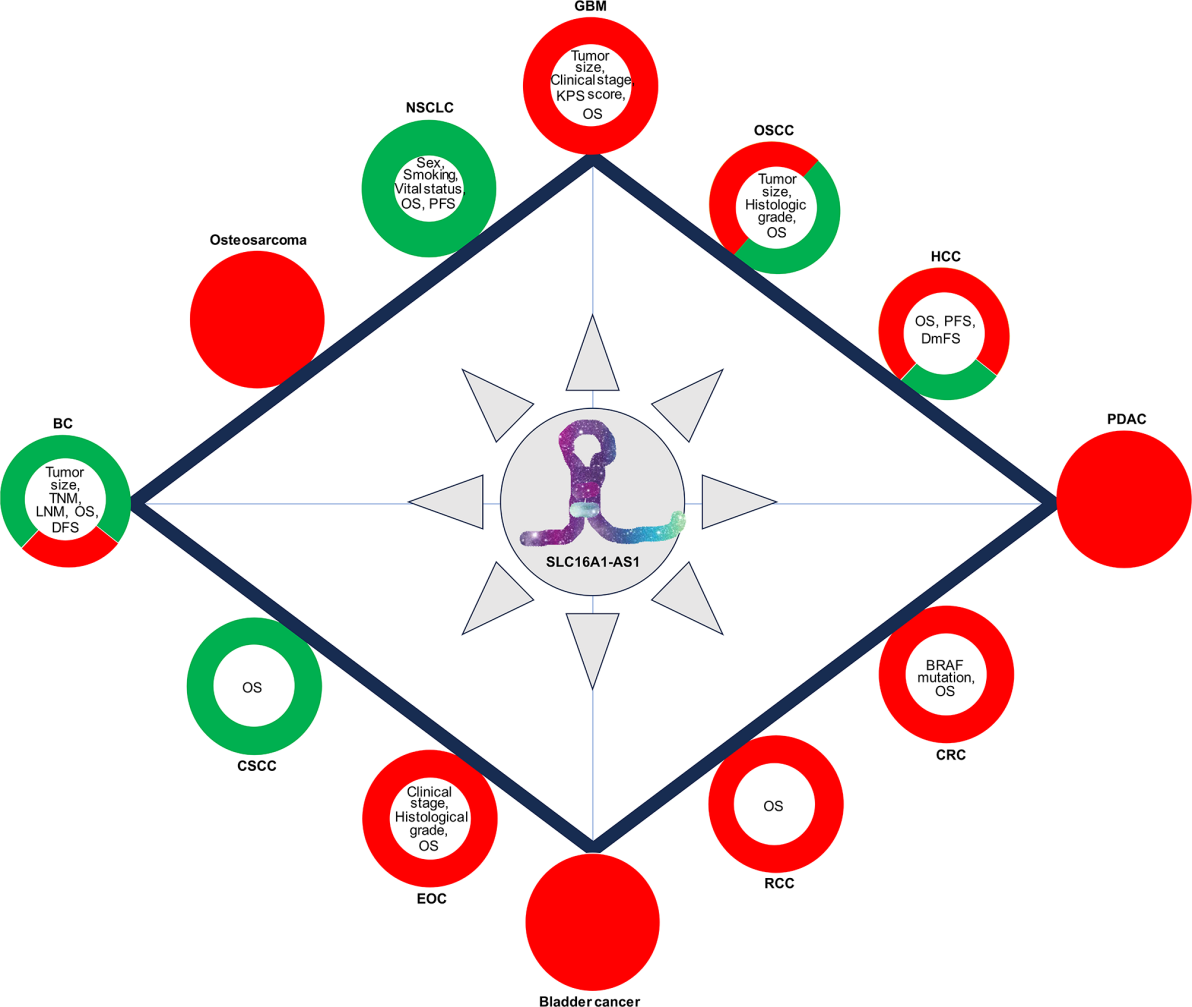
The Expression and Prognostic Network of SLC16A1-AS1 in Cancer. At the center of the diagram is SLC16A1-AS1, surrounded by a constellation of colored circles that represent its expression levels and clinical impact in various cancers. Red circles signify the upregulation of SLC16A1-AS1, while green circles indicate downregulation. The color variation of these circles not only reflects the expression levels of SLC16A1-AS1 but also underscores its prognostic significance in the oncological context

It has been reported that dysregulation of SLC16A1-AS1 is associated with clinical features and prognosis in various types of tumors, as summarized in Fig. 3. Notably, the prognostic value of SLC16A1-AS1 may vary across different tumors and clinical stages. Therefore, the prognostic significance of SLC16A1-AS1 in diverse tumor types warrants further investigation through larger clinical cohorts at different cancer stages. Additionally, SLC16A1-AS1’s potential as a diagnostic marker, particularly for differentiating tumor tissue from normal tissue, is highlighted in some tumor types, such as bladder cancer [102]. However, its diagnostic efficacy in other tumor types requires more exploration. Importantly, up to now, the detection of SLC16A1-AS1 expression has been limited to tumor tissue samples. The presence of SLC16A1-AS1 in liquid biopsies, such as plasma and urine, in cancer patients remains largely unexplored. Emerging evidence suggests that some circulating lncRNAs, such as MALAT-1 [151, 152], and TUG1 [153, 154], exhibit promising diagnostic potential as non-invasive tools for cancer diagnosis. Therefore, investigating whether SLC16A1-AS1 can serve as a non-invasive tumor marker for early cancer diagnosis presents an intriguing research direction.The potential of SLC16A1-AS1 to predict disease progression, response to treatment, and patient outcomes offers substantial prospects for enhancing personalized cancer care. Integrating SLC16A1-AS1 expression profiles into clinical practice could significantly improve patient management strategies, resulting in more personalized and effective cancer treatments.

## Conclusion

In conclusion, the intricate nature of SLC16A1-AS1 as a long non-coding RNA highlights its pivotal role in cancer biology, where it functions as both an oncogene and a tumor suppressor across different cancer types. This duality underscores the complexity of cancer mechanisms and reflects on SLC16A1-AS1’s significant influence on crucial cellular processes such as proliferation, apoptosis, and metastasis. The potential of SLC16A1-AS1 to serve as a novel biomarker for cancer prognosis and diagnosis represents a breakthrough in oncology, offering promising avenues for the development of targeted therapeutic interventions. Its ability to modulate key pathways in cancer cells positions it as a valuable target for innovative treatments aimed at halting cancer progression and improving patient outcomes. Moving forward, focused research on SLC16A1-AS1 will be indispensable in unraveling its multifunctional roles and in leveraging its therapeutic and diagnostic potential to combat cancer more effectively.

## Data Availability

No datasets were generated or analysed during the current study.
